# Role of Happiness: Mediating Digital Technology and Job Performance Among Lecturers

**DOI:** 10.3389/fpsyg.2021.593155

**Published:** 2021-02-26

**Authors:** Yuni Ros Bangun, Adita Pritasari, Fransisca Budyanto Widjaja, Christina Wirawan, Anggara Wisesa, Henndy Ginting

**Affiliations:** ^1^School of Business and Management, Institut Teknologi Bandung, Bandung, Indonesia; ^2^Industrial Engineering Department, Universitas Kristen Maranatha, Bandung, Indonesia

**Keywords:** digital technology, happiness, job performance, lecturers’ performance, oxford happiness questionnaire, work happiness

## Abstract

**Purpose:**

Happiness has been the most important goal for humans throughout history and is a significant issue among university lecturers facing a rapid digital technology change. It is usually described as a well-being state, feeling satisfied and contented, consisting of positive happenings in an individual’s life concerning the social, spiritual, economic, psychological, and physiological spheres. This research examines the relationship between happiness, attitudes toward technology, and lecturers’ job performance in higher education.

**Design and Methodology:**

This research design was a cross-sectional design that asked the respondents from lecturers of Institut Teknologi Bandung, one of the best universities with technology-based education in Indonesia, to complete a group of well-validated questionnaires. The questionnaires mentioned earlier include the Oxford Happiness Questionnaire and three other newly constructed questionnaires, made to measure attitude toward digital technology, job satisfaction, and job performance.

**Findings:**

This research confirmed that happiness fully mediated the relationship between attitude toward digital technology and job performance. Additionally, this research also confirmed that happiness partially mediated the relationship between job satisfaction and job performance. These results implied that a positive attitude toward digital technology and higher job satisfaction would lead to happier lecturers who increase their job performance.

**Originality:**

This study suggests that a positive attitude toward technology has a higher impact than job satisfaction as determinant factors of happiness and its association with lecturers’ job performance such as universities, especially Institut Teknologi Bandung as a technologically advanced workplace environment. Additionally, the Oxford Happiness Questionnaire framework, frequently used in studies of other countries, is now being used in the context of an Indonesian case study, precisely to measure happiness among lecturers in Indonesian higher education.

## Introduction

Recently, happiness has become a popular topic with the increasing concern for people’s importance in establishing the organization’s success. Happiness affects organizational performance (e.g., Warr, 2007; Fisher, 2010; Demircioǧlu, 2014; Santoso and Kulathunga, 2016). Zhou and Qiu (2013) stated that in a company, happiness, enthusiasm, initiative, and creativity are essential things to be considered when implementing people-oriented management. Despite being a problematic term to define, happiness has been the most crucial goal for humans throughout history (Compton, 2005). Aristotle (2012) believed everyone would agree that the term “eudaimonia” (happiness) was apt for such a state, which he regards as a metric for living well, central to human existence and purpose (Heeks, 2012). The pursuit of happiness is the ultimate goal in human existence (Pishva et al., 2011).

Happiness is usually described as a well-being state, a stable state of being well, feeling satisfied, and content. It consists of positive happenings in an individual’s life concerning social, spiritual, economic, psychological, and physiological spheres (Choudhury and Barman, 2014). Moreover, according to Seligman (2011), well-being is associated with happiness related to a pleasant, harmonious, and meaningful life. Defining happiness is challenging because its meaning in everyday usage seems very obvious (Bartram, 2012). Many researchers use data from surveys to measure happiness, where people are asked to indicate their happiness on a scale. Qualitative studies on happiness are less common because gaining data on people’s perceptions about happiness determinants might not be a reliable way to learn what leads to happiness (Bartram, 2012).

Speaking about the state of well-being, we have to admit that technology has become part of our daily life, signifying the current era of digital technology (O’Brien, 2016), including how it is related to happiness. This relationship between happiness and digital technology has become a perennial subject about how happiness involves a more capacious relationship between broad material prosperity and well-being (Surowiecki, 2005) and the impacts that digital technology brings to that well-being (Barman and Choudhury, 2014). Digital technology has introduced a slight beneficial impact on people’s sociability, connections with others, and sense of well-being, just as O’Brien (2016) said that well-being embraces the appropriate use of the broad continuum of technology.

Digital technology itself is one dimension that generates meaning, motivation, and effort in a person’s working life (Watson, 2008). For the adults who spend time mostly at work, work has affected health and happiness (Warr, 2007), including a person’s working life as a lecturer. Like other aspects of human life, digital technology has pervaded education institutions, not only the methods in the learning process (Sappey and Relf, 2010; Gallardo Echenique et al., 2015) but also the impacts on the well-being as well as job satisfaction of the workers and their performance. Nowadays, lecturers are expected to adapt to digital technology development, which could be the source and obstruction of happiness (Kavetsos and Koutroumpis, 2011). Ginting et al. (2020) confirm that digital technology has a significant impact on happiness. With the attitude toward digital technology, job satisfaction might also be associated with happiness, as it is a pleasurable or positive emotional state resulting from the appraisal of one’s job and job experiences (Tang et al., 2019). Happy workers should also report high levels of job satisfaction.

The determinants of happiness might differ between people and between organizations (Zhou and Qiu, 2013). This study elaborates on the relationship between happiness, attitude toward digital technology, job satisfaction, and job performance among lecturers in a higher education institution. Besides attempts to confirm the relationship between those variables, as mentioned earlier, this study also investigates the mediating role of happiness in the relationship between variables. Similar research about lecturers has never been done in Indonesia. The happiness research usually focused on the predictors, levels, and outcomes of happiness (e.g., Gunawan and Wahyuni, 2017; Batubara and El Hami, 2018; Gunawan and Alimin, 2018). In higher education institutions, digital technology is implicitly associated with improved happiness that leads to improved job performance (Ginting et al., 2020). A substantial gap in happiness and technology research emerges from the literature’s limited focus on its relationship. This study aims to fill in the gap by identifying lecturers’ happiness and their mediating roles in the relationships between attitude toward digital technology and job satisfaction.

## Literature Review

### Defining Happiness

Happiness is described as a stable state of well-being, feeling satisfied and content. It is a state that consists of positive happenings in an individual’s life concerning his social, spiritual, economic, psychological, and physiological spheres. It is a complex combination of a person’s life that is “how we feel about ourselves and our lives” (Choudhury and Barman, 2014).

There are two approaches in defining happiness as well-being: one focuses on subjective well-being and the other on psychological well-being. Subjective well-being is understood as having an affective (emotional) component, the balance between positive and negative effects, and a cognitive component of judgments about one’s life satisfaction. Psychological well-being is defined as “engagement with existential challenges of life” (Keyes et al., 2002).

### Lecturer’s Happiness

As work becomes less distinct and physically separated from other aspects of human lives, work will become increasingly entangled in a pure sense of identity: both as individuals and as citizens. The higher level of happiness expresses a strong sense of identity. Identity as a happy lecturer in the university might be represented by the sense of the job’s contribution to the civic society. Meanwhile, low happiness among lecturers might see their job as something they can easily disconnect from Albano (2009). These could be explained by Kjerulf (2014) finding that only 10% of happiness at work depends on the job itself, whereas 90% depends on the individual. The lecturer mostly influences the sense of identity as an individual to make meaning for the job, which might, in turn, impact the level of happiness. Ginting et al. (2020) suggested interpersonal and intrapersonal factors that determined the high level of happiness. Those factors were pleasant, good life, freedom, and the opportunities to share knowledge and values. Additionally, Arora (2020) also suggested that fringe benefits, personal growth, job security, salary, and social endeavors are factors impacting a lecturer’s happiness (Arora, 2020). In sum, happiness can cultivate a positive teaching and learning environment for both students and lecturers (Applasamy et al., 2014), which will bring an increased performance for lecturers.

### Digital Technology and Performance

The emergence of digital technology has become part of the daily life of today’s people everywhere. Despite its pros and cons, the use of digital technology is ever-growing and expanding. The use of digital technology in the industry has become commonplace, used extensively, and, in an increasingly sophisticated fashion, providing many benefits. The use of digital technology in manufacturing, for example, helps to improve productivity, quality, efficiency, monitor and reduce risk, and reduce the effects of human factors (Jose et al., 2016). In the hospital, digital technology usage was positive and significantly affected quality and revenue (Devaraj and Kohli, 2003). The advancement of information technology’s ability to present useful and broad information also benefits and improved marketing performance through efficient product placement and price dispersion reduction (Jensen, 2007). Performance in marketing is also increasing with e-commerce that allows trading activities carried out between regions efficiently. Here, digital technology helps to increase consumers’ and producers’ welfare (Jensen, 2007).

In the field of education, digital technology also has a broad impact through its use. Digital technology positively and negatively influences the learning process, student learning, and social life. Education institutions also attempt to use digital technology to raise the efficiency and effectiveness of the education process. Digital technology can be used to deliver the learning material and administration work, such as monitoring absenteeism, students’ and teachers’ performance, etc. (Underwood, 2009). Digital technology can also help teachers improve their performance by creating creative and interactive material that can help students learn, support research processes that will hopefully raise the research quality, and help in administrative work. According to Underwood (2009), digital technology in education results in a distinct change in behavior and performance. Behavioral change is related to increased learning readiness by learning the material to be accessed by the student before class. Regarding digital technology usage in education, change in performance is also expected if it is well-aligned with learning material (Higgins et al., 2012).

### Technology and Happiness

Nowadays, digital technology is prevalent in day-to-day life. Digital technology and well-being come hand in hand to create a cyclical co-production of one another (Choudhury and Barman, 2014). Choudhury and Barman mentioned four well-being parameters: psychological well-being, physical well-being, social well-being, and spiritual well-being. Technology has influenced all industries and has created a new way of living. The positives that technology brings also come to its negative attributes to society, affecting the individual’s well-being. Access to digital technology is favorable for one’s well-being in general terms, but there are also some negative ramifications for those whose access to technologies is relatively new (Graham and Nikolova, 2013).

Attitude toward digital technology is based on individual behaviors toward the need and digital technology in their work. The attitude itself is the representation of an individual’s emotional response toward something. The three aspects of attitudes are an individual’s emotional response, factual knowledge, and overt behavior directed toward some stimuli related to digital technology. In this research, these three aspects were used to construct the attitude model questionnaire (Rosenberg and Hovland, 1960; Eagly and Chaiken, 1993). The aspects of digital technology cover findings and practical utilities on devices, methods, and computerized systems, such as computer technology, the internet, digital communication, and social media. In this research, the attitudes toward digital technology are classified as a cognitive aspect (e.g., *I have a good skill in digital technology*), an affective aspect (e.g., *Digital technology, like the internet, scared me*), and a conative aspect (e.g., *I use digital technology even for a sensitive thing, such as buying medicines*).

### Happiness and Job Performance

For organizations, happiness is crucial (Hales and Williamson, 2010) because it improves employees’ productivity and job performance (Fisher, 2010; Rodríguez-Muñoza and Sanz-Vergel, 2013; Simmons, 2014) and, in turn, provides advantages to organizations. Research shows that employees tend to be productive in their work performance, improve effectiveness, and their creativity and innovativeness (Gupta et al., 2012; Wesarat et al., 2014). This condition shows how close the relationship is between happiness and workplace success. Moreover, Alipour et al. (2012) mention that happy employees achieve goals and challenges at a higher rate, less absent-minded, show increased job satisfaction, and are more efficient and motivated than those who are not happy. The most efficient way to increase productivity is to be happy to work better (Gupta, 2012). Those all explain why happy employees are relatively more successful and perform better (Lyubomirsky et al., 2005; Boehm and Lyubomirsky, 2009) and, in the end, contribute to the success of their organizations. Employee happiness is critical for achieving organizational success (Page and Vella-Brodrick, 2009).

We can see the same findings in educational institutions. Various research shows that happiness significantly drives work performance in educational institutions (Abedi et al., 2010; Field and Buitendach, 2011; Usop et al., 2013; Jalali and Heidari, 2016). Gholami et al. (2013) found a significant and positive relationship between an educational institution’s employees’ happiness and job performance. Seligman et al. (2009) observed that happiness has proven to alter success in the classroom effectively. On top of that, happiness boosts the performance of the students and teachers. Otherwise, teachers with low levels of happiness tend to be more exposed to job emotional capture (Seligman, 2011).

In a higher education context, we understand that a lecturer’s job demands are not simple. This situation is a claim supported by substantial research. For example, Chotivanich (2017) concluded five lecturer workloads: teaching, research, academic activities, academic outreach, cultural, and others. Lecturers must teach classes, conduct research and other academic activities, promote community well-being, and integrate cultural demands in their activities. Besides, some lecturers have to manage these tasks while acting as dean, head of a department, or other structural positions. Despite the individual’s calling to teach, the other demands can prove a burden for lecturers. Ginting et al. (2020) confirmed that these Institut Teknologi Bandung (ITB) lecturers defined success that creates happiness as when they could provide for their family, help students to excel, and when students can use the knowledge they taught. Ginting et al. (2020) confirm that it was a meaningful impact on happiness in work.

**FIGURE 1:**
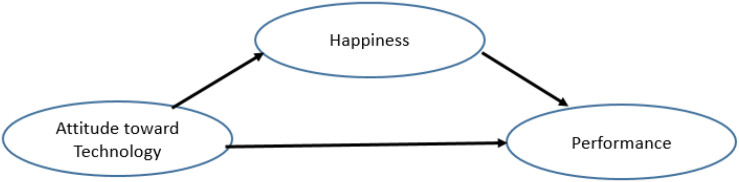
Hypothesis 1 happiness as a mediator between attitude toward digital technology and performance.

In this study, we hypothesized that happiness is the mediator between attitude toward digital technology and performance (see Figure 1). Attitude, skill, and knowledge are essential individual factors that influence job performance. Attitude toward digital technology as an essential issue in this era may be one of the influential factors to higher individual job performance. Digital technology is one of the leading standard equipment to support lecturers’ jobs. Even the most skilled and talented lecturers might be prone to severe underperformance if their digital technology attitude lacks. On the other hand, lecturers who have a positive attitude toward digital technology would enjoy and be satisfied with their jobs, leading to higher performance.

**FIGURE 2:**
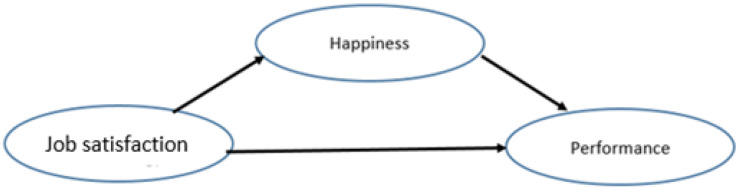
Hypothesis 2 happiness as a mediator between job satisfaction and job performance.

We also hypothesized that happiness is the mediator between job satisfaction and job performance (see Figure 2). It is widely recognized that professionals, including lecturers who have a positive attitude, are more productive and useful to the organization. One of the primary factors in lecturer attitude is job satisfaction. If lecturers enjoy their work, feel confident in their abilities to succeed in doing their tasks, and appreciate their role, they are far more likely to feel happy in the workplace. Lecturers who are satisfied with their job would be happier, which leads to higher performance. We focus on lecturers’ performance in three aspects: behavior that meets the standard, working results, and job responsibilities. Those three aspects were aligned with seven lecturers’ performance dimensions stated by Molefe (2010) and Viswesvaran et al. (2002). The questionnaire to measure job satisfaction involved four aspects: identity, opportunity to share, creativity, and encouragement (Tang et al., 2019).

## Materials and Methods

### Participants

This study was conducted at ITB (Bandung Institute of Technology), one of Indonesia’s best universities. The sample consisted of 126 ITB lecturers, most of them being married (88.1%), men (68%), Javanese (71.4%), and having a doctorate (73%). The demographic and personal characteristics of the participants are presented in Table 1. We sent an email to all lecturers in ITB, asking them to complete a set of online questionnaires voluntarily. Of 1,200 lecturers, only 126 lecturers responded and completed the questionnaire. This 10% response rate was achieved in 30 days. The criteria for eligible participants were full-time lecturers listed on the ITB’s School and Faculties websites. As for ethical consideration, the Research Institution of ITB has reviewed and approved the study procedures. All participants provided online, informed consent.

**TABLE 1:** Characteristics of participants.

Faculty/school*	Qty (%)	Academic level	Qty (%)	Annual income (IDR)	Qty (%)
Applied social sciences	36.5	Academic assistant	23.0	<60M	10.3
Natural sciences	17.5	Lecturer assistant	11.9	60–150M	49.2
Civil engineering	17.5	Assistant professor	45.2	>150–350M	31.7
Industrial engineering	28.5	Associate professor	10.3	>350–500M	7.1
		Full professor	9.6	>500M	1.6

*Applied Social Sciences (School of Business and Management, Faculty of Art and Design); Natural Sciences (Faculty of Mathematics and Natural Sciences, School of Pharmacy, Faculty of Earth Sciences and Technology); Civil Engineering (Faculty of Civil and Environmental Engineering, School of Architecture, Planning, and Policy Development); Industrial Engineering (Faculty of Industrial Technology, Faculty of Mechanical and Aerospace Engineering, Faculty of Mining and Petroleum Engineering, School of Electrical Engineering and Informatics); M = Million.

### Measures

This study has four main variables: attitude toward digital technology, happiness, job satisfaction, and job performance. Four questionnaires were used to measure those main variables: the Attitude Toward Digital Technology Questionnaire, the Oxford Happiness Questionnaire (OHQ), the Job Satisfaction Questionnaire, and the Perceived Job Performance Questionnaire.

The Attitudes Toward Digital Technology Questionnaire was constructed using a three-component attitude model (Rosenberg and Hovland, 1960; Eagly and Chaiken, 1993). Three aspects of attitudes are predispositions or representations of an individual emotional response or liking, factual knowledge, and overt behavior directed toward some stimuli related to digital technology. The aspects of digital technology cover findings and practical utilities on devices, methods, and computerized systems, such as computer technology, the internet, digital communication, and social media. Six questions from the Likert scale were used to capture those three aspects of attitudes toward digital technology. An exploratory factor analysis using maximum likelihood with varimax rotation showed that the cumulative variance was accounted for 68.93% (factors loading ≥0.3). It indicates that the three factors model (eigenvalues >1) represents the factorial validity of the three aspects of attitudes (Arrindell and Van der Ende, 1985). The first two questions about attitudes toward digital technology measured the cognitive aspect (e.g., *I have a good digital technology skill*), with a factor loading of 0.64 and 0.69 in the exploratory factor analysis. The third and fourth questions measured affective aspects (e.g., *Digital technology, like the internet, scared me*) with factor loadings of 0.70 and 0.39. The fifth and sixth questions measured conative aspects (e.g., *I use digital technology even for a sensitive thing, such as buying medicines*) with factor loadings of 0.38 and 0.45. Cronbach’s alpha for those six questions was 0.65, which reflects the questionnaire’s reliability.

The Oxford Happiness Questionnaire (Hills and Argyle, 2002) was used to measure happiness in this study. The OHQ has been proven to be consistent across cultures (Hills and Argyle, 2002) and to have good reliability and validity, providing a good analysis of happiness’s internal and external factors (Lu and Shih, 1997). In this study, the original English versions of the OHQ were translated into the Indonesian language using the forward–backward translation method (Guillemin et al., 1993; International Test Commission, 2010). Two qualified translators translated the original questionnaires to the Indonesian language. A native English speaker living in Indonesia for more than 20 years translated back from the Indonesian language into English. A committee of experts in clinical psychology reviewed the original English version, English back translation, and the Indonesian version of the questionnaire to produce a final translation. The final translation of the OHQ used in this study showed adequate internal reliability, with Cronbach’s alpha = 0.90. The principal components analysis of the present study’s data extracted seven factors with eigenvalues >1, which accounted for 63.58% of the respective total variances. The results showed factorial validity of the translated OHQ, which slightly confirmed the eight factors of the original OHQ (Hills and Argyle, 2002).

The Job Satisfaction Questionnaire was constructed using Tang et al. (2019). They defined job satisfaction, based on their literature perspectives, as the perceived agreement of employee’s expectations with their actual work process, responsibilities, and job context. It is an overall feeling or affective response and the employee’s perspective regarding their job and organization. To measure those overall feeling and perspective, we constructed four items: identity (i.e., *Being a lecturer in ITB is an honored identity for me*), the opportunity to share (i.e., *I have an opportunity to share values and meanings during lecturing in ITB*), creativity (i.e., *I have freedom in ITB to be a creative lecturer*), and encouragement (i.e., *My contributions in ITB are accepted and encouraged*). The questionnaire’s inter-item correlations were between 0.47 and 0.63, which might reflect validity, and Cronbach alpha was 0.84, which reflected high reliability.

The Perceived Job Performance Questionnaire referred to Viswesvaran et al. (2002). They defined job performance as scalable responsible actions, behavioral standards, and results or outcomes that employees engage in which align with organizational goals. Viswesvaran et al. (2002) also suggested various methods to measure job performance, including rating scales from self and others and organizational records that might suffer from criterion bias. Hence, in this study, we used self-report (rating), which has greater criterion relevance because the participants have better knowledge and motivation to evaluate their performance. The Questionnaire of Perceived Job Performance consisted of three items, which referred to Viswesvaran et al. (2002). Those items measured lecturers’ behavioral standards (i.e., My behaviors meet ITB standard), result or outcome (i.e., My working results meet ITB requirement), and responsibilities (i.e., Up and down of ITB is part of my responsibility). The questionnaire’s inter-item correlations were between 0.36 and 0.72, which might reflect their reliability, and the Cronbach alpha was 0.73, which reflected high reliability.

### Data Analysis

All statistical analyses were performed using SPSS 21 software (IBM). All omnibus tests were considered statistically significant if *p* < 0.05. Descriptive statistics were provided as mean and standard deviations for continuous variables and percentages for categorical variables. Pearson correlations were used to examine univariate associations between continuous variables and point-biserial correlations for dichotomous variables.

Two mediation analyses following Baron and Kenny’s criteria for mediation (1986) were conducted through a series of hierarchical regressions to test the mediating role of happiness (total score of OHQ) in the association between attitudes toward digital technology and job satisfaction with perceived performance. In these analyses, digital technology attitudes in the first model or job performance in the second model were entered as the independent variables. In contrast, happiness was entered as the mediator, and job performance was entered as the dependent variable. Demographic and personal characteristics (i.e., sex, income, educational level, ethnicity, academic level, faculty/school, and marital status) served as the control variables. The attitudes toward digital technology scores, the OHQ scores, the job performance scores, and job satisfaction scores were mean-centered before conducting the mediation analysis. The assumption for the analysis (normal distribution of residuals) was tested using a normal probability plot. There were no indications of the violation of this assumption. Furthermore, correlations between independent variables and tolerances were calculated to check for multicollinearity. All tolerance values were between 0.45 and 0.88, and all correlations were less than 0.64.

According to Baron and Kenny (1986), mediation is demonstrated when the following conditions are met: (1) the independent variables (i.e., attitudes toward digital technology or job satisfaction) affect the mediator (i.e., happiness); (2) the independent variables affect the dependent variable (i.e., perceived performance); (3) the mediator affects the dependent variable when the independent variable is controlled for; and (4) full mediation is confirmed when the association between the independent variable and dependent variable is reduced to non-significance after the effect of the mediator is controlled. If conditions 1–3 are met, partial mediation is indicated.

## Results

### Mediating Role of Happiness in the Relation Between Digital Technology and Performance

A separate regression analysis showed that digital technology attitudes significantly predicted happiness, indicating that condition 1 for mediation was met, β = 0.45, *t*(124) = 2.92, *p* < 0.001. A first hierarchical regression analysis was conducted with attitude toward technology as an independent variable, happiness as a mediator, and job performance as a dependent variable. At step 1 of this hierarchical analysis, digital technology’s attitude was a significant predictor of job performance, β = 0.25, *t*(124) = 5.61, *p* = 0.005, indicating that condition 2 for mediation was met. Condition 3 was met because when happiness entered the equation at step 2 of the hierarchical regression, it significantly predicted job performance, β = 0.22, *t*(124) = 2.34, *p* = 0.021, and reduced the beta weight for attitude toward digital technology to non-significance, β = 0.15, *t*(124) = 1.60, *p* = 0.133. Because condition 4 was met, it showed that the relationship between attitude toward digital technology and job performance was fully mediated by happiness.

### Mediating Role of Happiness in the Relation Between Job Satisfaction and Job Performance

Next, we investigated the possible mediation of the relation between job satisfaction and job performance by happiness. A separate regression analysis showed that job satisfaction significantly predicted happiness, indicating that condition 1 for mediation was met, β = 0.64, *t*(124) = 8.64, *p* < 0.001. A first hierarchical regression analysis was conducted on job satisfaction as an independent variable, happiness as a mediator, and job performance as a dependent variable. At step 1 of this hierarchical analysis, job satisfaction was a significant predictor of job performance, β = 0.55, *t*(124) = 6.98, *p* < 0.001, indicating that condition 2 for mediation was met. Condition 3 was met because when happiness entered the equation at step 2 of the hierarchical regression, it significantly predicted job performance, β = 0.35, *t*(124) = 3.38, *p* = 0.001, and reduced the beta weight for attitude toward digital technology, β = 0.31, *t*(124) = 3.05, *p* = 0.003. Because condition 4 was not met, it showed that the relationship between attitude toward digital technology and job satisfaction was partially mediated by happiness.

### Association Between Attitude Toward Digital Technology and Happiness

Table 2 below shows the multiple regression analysis results, which tested associations between demographic and personal characteristics and the three aspects of digital technology attitudes with happiness.

**TABLE 2:** Multiple regression analysis on attitudes toward digital technology and happiness, controlled for demographic and personal characteristics.

Predictive variable	Happiness	
	
	Step 1 (β)	Step 2 (β)
Demographic/Personal Characteristics	*R*^2^ = 0.08*	*R*^2^ = 0.21**
Sex	0.12	0.13
Faculty/School		
Applied social sciences	0.21*	0.10
Natural sciences	−0.28*	–0.15
Civil engineering	–0.20	–0.16
Industrial engineering	–0.06	–0.09
Marital status	–0.08	0.01
Ethnicity	–0.01	0.03
Academic level	0.15	0.20
Educational level	0.03	–0.03
Income	0.31	0.08
Cognitive Aspect		0.28**
Affective Aspect		0.05
*Conative (Behavioral) Aspect*		0.20*

*p ≤ 0.05; **p ≤ 0.01.

In step 1 of regression analysis, sex, income, educational level, ethnicity, academic level, faculty/school, and marital status were entered as independent variables and happiness as the dependent variable. The variance explained in step 1 was significant (*R*^2^ = 0.08, *p* = 0.04) in which the participants from applied social sciences were associated with higher happiness (β = 0.20, *p* = 0.04). Others, especially the natural sciences participants, were associated with lower happiness (β = 0.28, *p* = 0.01).

After adding the three aspects of attitudes toward digital technology in step 2, all demographic and personal characteristics were no longer significantly associated with happiness. The variance explained in step 2 was significant (*R*^2^ = 0.21, *p* < 0.001) in which both of cognitive and conative aspects were positive significant predictors (β = 0.28, *p* = 0.007 and β = 0.20, *p* = 0.046, respectively) of happiness but not affective aspect (β = 0.05, *p* = 0.63).

These results suggest that demographic or personal characteristics have a minimal effect on individuals’ happiness. However, their attitudes toward digital technology, especially their knowledge and behavior, would influence their happiness level. Affective aspects, such as emotional feeling toward digital technology, were not associated with happiness.

## Discussion

This research confirms happiness’s conceptual hypothesis model as a mediator of attitudes toward technology to performance and a mediator of job satisfaction to performance. It means the role of happiness is essential to increase lecturers’ job performance.

The association between attitudes toward digital technology and happiness shows that from the three elements of “Attitudes Toward Technology,” only cognitive aspects and conative aspects are associated with happiness, whereas affective aspects were not. It explains that lecturers’ knowledge is related to happiness and lecturers’ behavior, whereas digital technology’s emotional factor is not. *It can be explained that using technology in doing lecturers’ job is one of the main responsibilities to support their job effectiveness and efficiency and nothing to do with the emotional part (“Digital technology, like the internet, scares me.”)*. This finding was aligned with Underwood (2009) and Higgins et al. (2012) in their study, which explains using technology to increase lecturers’ performance.

This research confirms that happiness has partially mediated the relationship between job satisfaction to performance. It means that happier lecturers will have higher job satisfaction. This research also confirms that job satisfaction has an impact on happiness. This finding was supported by Ginting et al. (2020) in their study, which explains that job satisfaction has impacts on happiness.

Our research’s performance statements are developed by lecturers’ performances: identity, opportunity to share knowledge, creativity, and encouragement. This research confirms to say that attitude toward technology (knowledge and conative), mediating by happiness, will influence lecturers’ performance behavior in terms of identity, opportunity to share knowledge, creativity, and the encouragement to do the lecturers’ job.

Some aspects that might affect the lecturer’s happiness, which was not recorded in this research, are age and personality that, according to some studies, might have a relationship. Oswald et al. (2015) found that age might have an impact on the level of happiness. Another study found that relation age shows curvilinear relations with happiness (e.g., Pressman and Cohen, 2005; Veenhoven, 2008; Tadic et al., 2013). This finding somehow could be explained by some studies in the psychological area. For example, Morgan et al. (2015) said that older people tend to attend more favorable information, which implies that happiness would be more stable or increase with age.

Another aspect that was found to be a factor affecting people’s degree of happiness is their personality. A study among academicians in Malaysia (Aziz et al., 2014) has shown that most of them are at a medium level in terms of the affective and cognitive state throughout their lives. They suggest that happiness has been influenced by personality traits such as extraversion, agreeableness, conscientiousness, and openness but not neuroticism. This statement was in line with (DeNeve and Cooper, 1998; Aziz et al., 2014) statement, who said that personality factors play an important role in happiness.

The findings in this research show that there is no relationship between sex and people’s happiness. This result is supported by Sharif et al. (2012 in Aziz et al., 2014), who found that happiness among academicians is affected significantly by health conditions but not by sex.

According to Wright and Cropanzano (2000), psychological well-being and job satisfaction work as predictors of job performance. They remain suitable predictors of job performance even when controlling for job satisfaction, age, sex, and tenure.

The last is the degree of employee engagement. Nielsen et al. (2008) and Bataineh (2019) said that one indicator of good mental health is engaging employees at work. An employee who feels good and deals with less at work and home is more likely to experience satisfaction toward their work, which can significantly affect their well-being and organization (Koubova and Buchko, 2013; Bataineh, 2019). Therefore, maintaining the lecturer’s engagement is also essential to improve their job performance and happiness level.

Academicians without happiness may feel sad, anxious, empty, hopeless, worthless, guilty, irritable, or restless. According to Sharif et al. (2012), happiness among academicians significantly impacts health conditions. Happier employees contributed more toward being motivated and productive and better at maintaining relationships in the workplace (Aziz et al., 2014).

## Conclusion

This research reinforces the relationship between lecturers’ attitudes toward digital technology, job satisfaction, happiness, and lecturers’ performance. The role of happiness is as a mediator in relating attitude toward digital technology to lecturers’ performance. This research confirmed that cognitive aspects and conative aspects in digital technology would influence happiness. This finding gives the practical implication that higher education institutions should be aware of their lecturers’ cognitive and conative aspects of digital technology. It also confirms that attitudes toward digital technology will influence how lecturers perceive their job performance.

Moreover, this research found that demographic or personal characteristics have a minimal effect on individuals’ happiness. However, their attitudes toward digital technology, especially their knowledge (cognitive) and behavior (conative), would influence their happiness level. Affective aspects, such as emotional, feeling, toward digital technology, was not associated with happiness. As for the study’s contributions, it can be determined that it is essential to manage higher education institutions to pay attention to how important it is to prepare their lecturers’ readiness toward rapid digital technology changes. Besides, they will have to pay more attention to lecturers’ happiness because it will affect their job performance.

This study has limitations that are fruitful for further research. First, this research only studied lecturers at ITB. All respondents were ITB lecturers. Expanding respondents will improve the quality of research results. Second, this study only used OHQ as a measure of happiness. OHQ is indeed quite widely used, standardized, and tested, but there is still the possibility that there may be other variables that have not been included in this measuring instrument.

## Direction for Further Research

In general, research on lecturers at other universities in Indonesia, private and public universities, and even abroad needs to be carried out and give more convincing results. Research on several universities also provides opportunities for researchers to make comparisons and then look for best practices, such as comparing lecturers at private and public universities and between lecturers at each geographic area. Next, this study only uses OHQ, which has been standardized and tested. A qualitative study to develop and test a questionnaire will help anticipate the possibility of different variables.

## Data Availability Statement

The original contributions presented in the study are included in the article/supplementary material, further inquiries can be directed to the corresponding authors.

## Ethics Statement

Ethical review and approval was not required for the study on human participants in accordance with the local legislation and institutional requirements. Written informed consent for participation was not required for this study in accordance with the national legislation and the institutional requirements.

## Author Contributions

All authors listed have made a substantial, direct and intellectual contribution to the work, and approved it for publication.

## Conflict of Interest

The authors declare that the research was conducted in the absence of any commercial or financial relationships that could be construed as a potential conflict of interest.
